# Impact of COVID-19 Pandemic on Routine Immunization in State of Kuwait: Short-Term Disruption With Rebound in Vaccination Utilization

**DOI:** 10.1016/j.focus.2022.100031

**Published:** 2022-09-12

**Authors:** Najla H.A. Al-Ayyadhi, Shaimaa Sh. N. Al-Awadhi, Radhia F.A. Al-Mathkouri, Eman B.A. Al-Tayar

**Affiliations:** Directorate of Public Health, Ministry of Health, Kuwait

**Keywords:** COVID-19, routine vaccination, impact, missed vaccination, rebound

## Abstract

**Introduction:**

This study aimed to explore the impact of the COVID-19 pandemic on routine immunization along 4 abbreviated time frames: before the pandemic in 2019, stay-at-home period (March–May) in 2020, reopening period (June–August) in 2020, and corresponding months in 2021.

**Methods:**

A secondary analysis of immunization data in Kuwait during the prepandemic period in 2019, stay-at-home period (March–May) in 2020, reopening period (June–August) in 2020, and corresponding months in 2021 was conducted. All vaccines given at 2, 3, 6, 12, 18, and 24 months of age were included in the study.

**Results:**

The mean of total visits from March 2020 to May 2020 dropped (−28.9%) compared with the visits in March 2019–May 2019 and then increased during the reopening period in June 2020–August 2020 (+31.8%). All vaccinations scheduled for children aged ≤24 months showed a reduction. The greatest reduction was detected at age 24 months (−44.2%), followed by age 18 months (−36.5%) and then age 1 year (−28.8%). There were greater declines among non-Kuwaiti children than among Kuwaiti children for all types of vaccines. The mean of total visits in March 2021–May 2021 increased (+15.4%) compared with the mean in the same period in 2020. However, a reduction of −16.0% still exists compared with the reduction at baseline in 2019.

**Conclusions:**

The COVID-19 pandemic had a large impact on childhood vaccinations, with recovery in subsequent months.

## HIGHLIGHTS

• This study analyzed immunization data in Kuwait during periods related to the pandemic.
• The mean of vaccination visits dropped (−28.9%) during lockdown compared with 2019.
• Recoveries in vaccination visits increased during the reopening period (+31.8%).
• Greater declines were seen among non-Kuwaiti children than among Kuwaitis for all types of vaccines.
• The pandemic had negative impacts beyond direct viral infection, jeopardizing immunization.

## Graphical abstract

**Figure:**
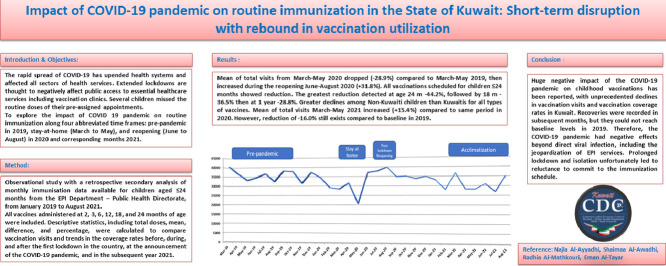

## INTRODUCTION

On March 11, 2020, WHO declared an international state of emergency to control the spread of severe acute respiratory syndrome coronavirus 2 (SARS-CoV-2), which started in Wuhan, China in 2019. It spread rather quickly across the globe causing the coronavirus disease of 2019 (COVID-19) pandemic.^1^ Currently, more than 500 million individuals have been affected worldwide with >5 million deaths.^1^^,^^2^ The introduction of SARS-CoV-2 in Kuwait from overseas was first reported on February 24, 2020.^3^

To control the spread of COVID-19, strict control measures were taken in most countries, including Kuwait, where preventive measures were initiated 2 days before confirming the discovery of the index case.^4^ Measures to mitigate the risk of the pandemic focused on the use of face masks, frequent hand washing, social distancing, following quarantine procedures, imposing partial or complete lockdowns with stay-at-home orders, closing of schools and religious places, working and studying from home, and preventing gatherings.

The people of Kuwait effectively adhered to a complete suspension of activities in the public sector and ministries beginning the third week of March. Some restrictions were eased from mid-April until a full lockdown was imposed from May 10, 2020 to May 30, 2020 across the country to curb the spread of the virus. Complete lockdown and movement restrictions were imposed in 2 areas for 3 months to prevent the spread of SARS-CoV-2 from April 6, 2020 to July 10, 2020. The second phase of easing the COVID-19 restrictions began in June 2020.^3^^,^^4^

The rapid spread of COVID-19 has upended health systems and affected all sectors of health services. Extended lockdowns are thought to negatively impact public access to essential healthcare services, including vaccination clinics. Several children missed the routine doses of their preassigned appointments. Globally, an estimated 13.5 million children missed routine vaccinations in 2020 because of the diversion of health activities and resources toward the control of newly discovered COVID-19 cases.^5^ Immunization is considered a cornerstone in preventing several fatal infectious diseases. The success of pediatric immunization programs depends on high uptake levels of vaccine doses at a particular time and age to achieve a significant decline in the incidence and prevalence of vaccine-preventable diseases (VPDs).^5^ However, scrupulous preventive measures, including lockdowns and social distancing, along with the fear of infection, possibly resulted in reduced provision, accessibility, and approachability of routine immunization services. The disruption of routine vaccine services could threaten children with contracting VPDs and initiate secondary outbreaks. A review by Lassi et al.^6^ suggested a potential 10% increase in mortality from VPDs owing to pandemic-associated disruptions to immunization. Previous outbreaks, such as the 2014 Ebola virus epidemic in West Africa, disrupted immunization programs and resulted in subsequent measles outbreaks^7^, ^8^, ^9^ and an increase in polio cases.^6^^,^^10^

In Kuwait, all vaccines are administered free of charge regardless of nationality or area of residence, that is, whether the individual lives inside or outside the primary healthcare center catchment area. Immunization is mandatory for children, adolescents, healthcare workers, food handlers, incoming expatriates, foreign students, and pilgrims (Appendix Table 1, available online). They are administered at vaccine centers throughout the country. The expanded program of immunization (EPI) system is integrated into other primary healthcare programs. The coverage levels are calculated annually on the basis of an administrative method and surveys measuring vaccine coverage at the district level every 5 years. All pediatric vaccines included in the EPI plan have had a coverage rate >98% at the national level and >95% at the subnational and district levels for the last 2 decades.^11^ The improvements made thus far in population immunity need to be sustained by maintaining high immunization coverage. Hence, this study was conducted to assess the impact of the COVID-19 pandemic on routine immunization by comparing immunization visits during 4 abbreviated time frames: prepandemic period in 2019, the period of the first lockdown imposed in the country with stay-at-home orders (March–May), the reopening period (June–August) in 2020, and the months in 2021 corresponding to these periods.

**Table 1:** Means and Total Vaccination Visits by Month March–August 2019, 2020, and 2021

Year and change over time	March	April	May	Mean March–May	June	July	August	Mean June–August
2019	37,285	33,007	29,947	33,413	31,128	32,995	29,266	31,130
2020	25,306	28,422	17,508	23,745	33,819	34,580	36,091	34,830
2021	33,802	25,024	25,419	28,082	28,326	24,876	32,004	28,402
Change 2020–2019, %	−32.1	−13.9	−41.5	**−28.9%** **(*p*=0.044)** **Cohen's d effect size=2.49**	+8.0	+4.6	+18.9	**+10.6** **(*p*=0.030) Cohen's d effect size=2.922**
Change 2021–2020, %	+25.1	−13.5	+31.1	**+15.4%** **(*p*=0.185)** **Cohen's d effect size=1.009**	−16.2	−28.1	−11.3	**−18.5** **(*p*=0.048)** **Cohen's d effect size=2.971**
Change 2021–2019, %	−9.3	−24.2	−15.1	**−16.0%** **(*p*=0.104)** **Cohen's d effect size=1.495**	−9.0	−24.6	+9.4	**−8.8** **(*p*=0.162)** **Cohen's d effect size=1.175**

*Notes:* Boldface indicates statistical significance (*p*<0.05). The mean change between lockdown (March–May) and reopening (June–August) period in 2020 is +31.8% (*p*=0.039). Cohen's d effect size=3.35.

## METHODS

### Study Population

Kuwait is a small country that hosts one of the most modern healthcare infrastructures in the Middle East. Healthcare services are provided by the government free of charge to all citizens as well as to non-Kuwaiti residents.

This was an observational study with a retrospective secondary analysis of monthly immunization data available for children aged ≤24 months from the EPI Department, Public Health Directorate from January 2019 to August 2021. Vaccine administration in the following 4 time periods was analyzed: March 2019–May 2019 (before pandemic), March 2020–May 2020 (lockdown or stay-at-home period), June 2020–August 2020 (post-lockdown period), and March 2021–May 2021 (acclimatization).

### Measures

All vaccines administered at ages 2, 3, 6, 12, 18, and 24 months were included in this study. The birth dose of hepatitis B virus vaccine was excluded from the analysis because most neonatal deliveries occur in maternity hospitals or wards in general hospitals. In the case of childbirth outside the hospital, the newborn should be taken to the maternity hospital for examination, registration, and vaccination. Thus, almost all live births are vaccinated on the first day of life even during the pandemic.

### Statistical Analysis

Monthly statistical data extracted from the EPI department included the number of vaccinated children according to age, nationality, and the type and dose of vaccine administered. Data sorting and cleaning were carried out, and the relevant variables were recorded using Microsoft Excel. The following variables were used in the final analysis: total monthly visits; Bacille Calmette–Guerin vaccine; Pentavalent vaccine (Diphtheria, Pertussis, Tetanus, Hepatitis B, and Hib); Rota vaccine; MenACWY vaccine; measles, mumps, and rubella (MMR) vaccines; inactivated polio vaccine; oral polio vaccine (OPV); pneumococcal conjugate vaccine (PCV); and varicella vaccine (VV). Descriptive statistics, including total doses, mean, difference, and percentage, were calculated and tabulated. Bar charts and line graphs were plotted to compare vaccination visits and trends in the coverage rates before, during, and after the first lockdown in the country; at the announcement of the COVID-19 pandemic; and in the subsequent year 2021. Student's *t*-test was used to compare the means for evaluating the change between different periods. The significance level was set at 5%, with *p*<0.05 indicating a significant difference. Given the nature of the available aggregated data, no adjustment for potential confounders or effect modifiers was possible. In addition, the effect size in terms of Cohen's d was calculated according to the formula d=(M1−M2)/SDpooled, where SDpooled=√[(SD12+SD22)/2], M1 is the mean of Group 1, M2 is the mean of Group 2, SD1 is the SD of Group 1, SD2 is the SD of Group 2, and SDpooled is the pooled SD.

## RESULTS

**Figure 1:**
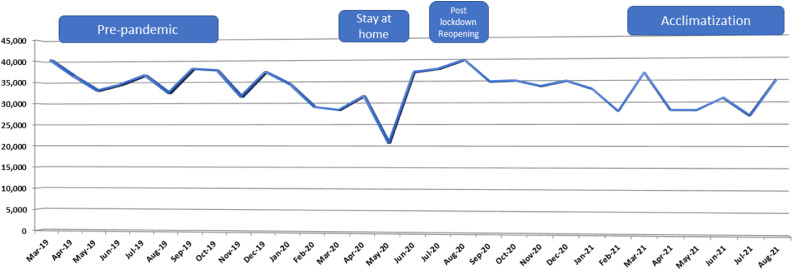
Vaccination visits for the primary vaccination series from Mar 2019 to Aug 2021.

The decline in visits for the primary series of vaccination in Kuwait started in November 2019. Then there was a distinct reduction during the lockdown period from March 2020 to May 2020 (Figure 1).

Apr, April; Aug, August; Dec, December; Feb, February; Jan, January; Jul, July; Jun, June; Mar, March; Nov, November; Oct, October; Sep, September.

The mean total number of visits for the primary series of vaccination during March 2020–May 2020 was reduced to 23,745 visits from 33,413 visits in March 2019–May 2019 (−228.9%) and Cohen's d=2.49, indicating a large effect size. This mean value corresponding to the period of March 2021–May 2021 increased to 28,082 visits, and the percentage change in March 2021–May 2021 compared with that of the same period in 2020 was +15.4%, with a Cohen's d=1.009 (large effect size). However, a comparison between 2021 and the baseline in 2019 still showed a reduction (−16.0%), with a Cohen's d=1.495 (large effect size) (Table 1).

**Figure 2:**
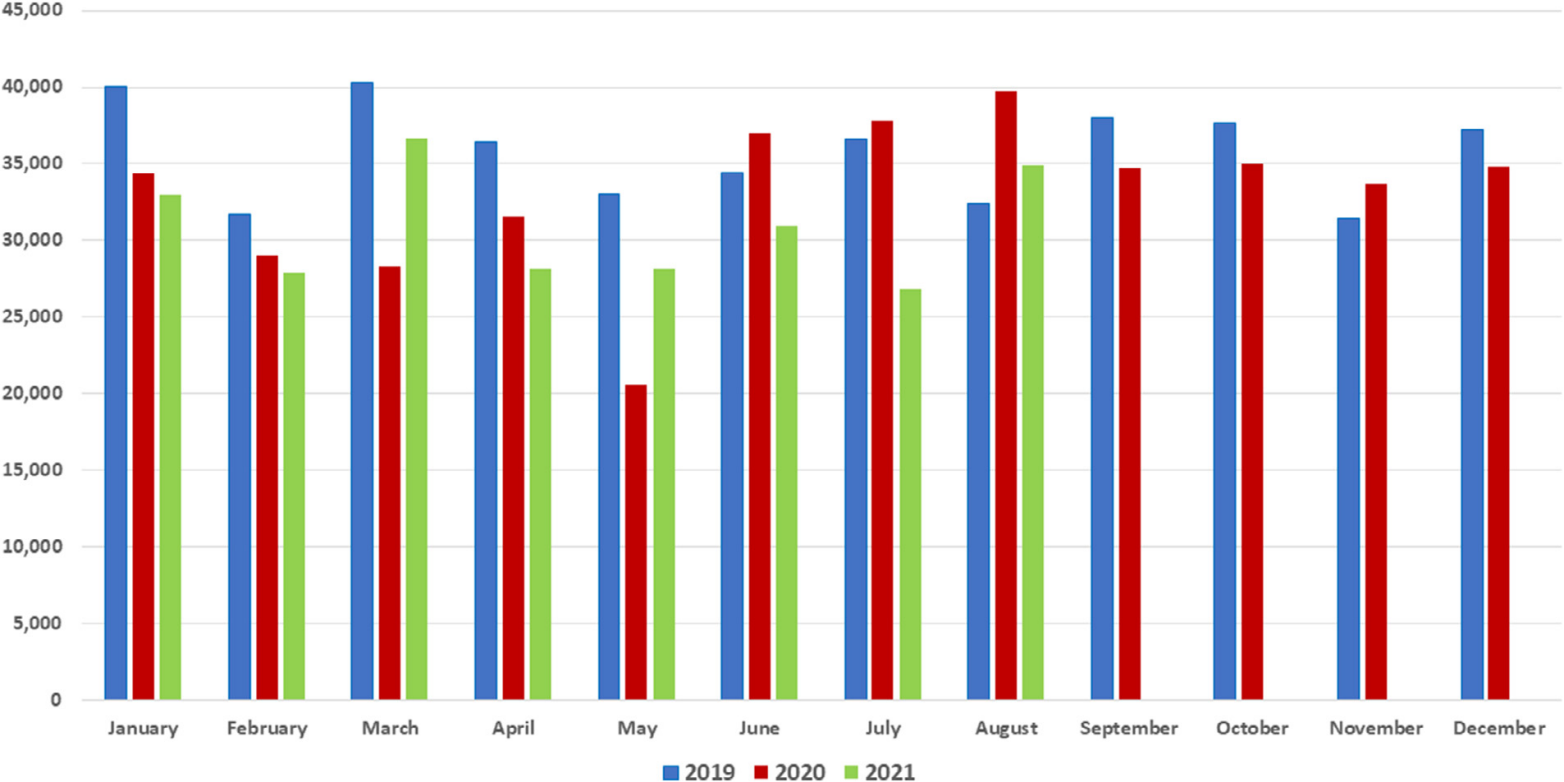
Comparison of vaccination visits for the primary vaccination series during 2019, 2020, and 2021.

The reduction in visits for the primary series of vaccination was relatively higher in March 2020 (−32.1%) than in April 2020 (−13.9%). The reduction was highest in May 2020 (−41.5%). Compared with the vaccination visits in the corresponding months in 2021, the number of vaccination visits increased mainly in March and May 2021 (+25.1% and +31.1%, respectively) (Table 1 and Figure 2).

The mean total number of visits during the reopening period from June 2020 to August 2020 increased to 34,830 with a +31.8% rebound from that during the lockdown period (March 2020–May 2020), with a Cohen's d=3.35 (large effect size). The total numbers of child vaccination visits were 31,128 and 33,819 in June 2019 and June 2020, respectively, showing an increase of +8.0%. The total number of visits in June 2021 was 28,326, representing a reduction of −16.2%; however, compared with the baseline in 2019, the reduction was −9.0%. Similar results were observed for July and August 2020 (Table 1 and Figure 2).

**Figure 3:**
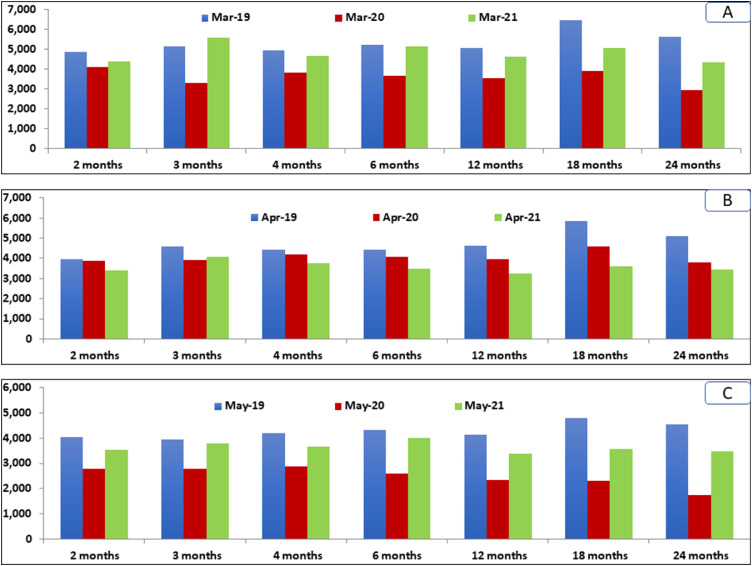
Vaccination visits for the primary vaccination series by age in the following months: (A) Mar; (B) April; and (C) May in 2019, 2020, and 2021.

All vaccinations scheduled for children aged ≤24 months showed a reduction in delivered vaccine doses in March 2020–May 2020. The greatest reduction was detected for vaccinations administered at age of 24 months (MMR2 and VV2) at −44.2%, followed by those at age of 18 months (Penta4, PCV4, and OPV2) at −36.5% and those at age of 1 year (MMR1 and VV1) at −28.8% (Table 2 and Figure 3A–C).

**Table 2:** Mean of Total Vaccinations by Age During March–May 2019, 2020 and 2021

	Vaccination at age, months	Mean		Reduction March–May 2019–2020, %	*p*-value	Mean March–May 2021	Change in vaccination mean	
		March–May 2019	March–May 2020				IncreaseMarch–May 2020–2021, %	Reduction March–May 2019–2021, %
Total Kuwait	2	4,280	3,592	−16.1	0.119	3,777	+4.9	−11.8
	3	4,561	3,347	−26.6	0.031	4,480	+25.3	−1.8
	4	4,522	3,632	−19.7	0.070	4,021	+9.7	−11.1
	6	4,665	3,443	−26.2	0.049	4,216	+18.3	−9.6
	12	4,607	3,278	−28.8	0.046	3,756	+12.7	−18.5
	18	5,694	3,617	−36.5	0.033	4,081	+11.4	−28.3
	24	5,084	2,835	−44.2	0.021	3,752	+24.4	−26.2
Kuwaiti	2	2,624	2,287	−12.9	0.145	2,490	+8.2	−5.1
	3	2,724	2015	−26.0	0.034	2,812	+28.3	+3.2
	4	2,740	2,251	−17.8	0.087	2,490	+9.6	−9.1
	6	2,765	2101	−24.0	0.056	2,603	+19.3	−5.8
	12	2,673	1979	−26.0	0.056	2,346	+15.6	−12.2
	18	3,253	2,213	−32.0	0.056	2,586	+14.4	−20.5
	24	2,844	1,720	−39.5	0.079	2,329	+26.1	−18.1
Non-Kuwaiti	2	1,656	1,305	−21.2	0.111	1,286	−1.5	−22.3
	3	1,837	1,332	−27.5	0.052	1,668	+20.1	−9.2
	4	1,782	1,381	−22.5	0.059	1,286	−7.4	−27.8
	6	1901	1,343	−29.4	0.037	1,612	+16.7	−15.2
	12	1934	1,299	−32.8	0.033	1,410	+7.9	−27.1
	18	2,441	1,404	−42.5	0.027	1,495	+6.1	−38.7
	24	2,240	1,114	−50.3	0.012	1,423	+21.7	−36.5

Apr, April; Mar, March.

The surge in the number of doses of the primary series of vaccinations during March 2021–May 2021 was the highest for vaccinations administered at the age of 3 months (Bacille Calmette–Guerin) at +25.3%, followed by those at age 24 months (MMR2 and VV2) at +24.4%. However, there was a reduction observed in comparing the vaccination of children aged ≤24 months in this period with that in the baseline period in 2019, especially at the ages of 18 months (−28.3%) and 24 months (−26.2%) (Table 2 and Figure 3A–C).

Vaccination of non-Kuwaiti children was lower than that of Kuwaiti nationals in 2020 for all vaccine types. For vaccinations administered at the age of 24 months, the reduction among non-Kuwaiti residents was −50.3%, whereas that among Kuwaiti nationals was −39.5%. For vaccinations administered at the age of 18 months, the reduction was −42.5% and −32.0% among non-Kuwaiti residents and Kuwaiti nationals, respectively. The increase from 2020 to 2021 for the same period was higher among Kuwaiti residents than among non-Kuwaiti nationals. Among the Kuwaiti children, the changes in vaccination doses at ages 2, 3, 4, 6, 12, 18, and 24 months were +8.2%, +28.3%, +9.6%, +19.3%, +15.6%, +14.4%, and +26.1%, respectively, whereas among the non-Kuwaiti children, the changes were −1.5%, +20.1%, −7.4%, +16.7%, +7.9%, +6.1%, and +21.7%, respectively (Table 2).

## DISCUSSION

In July 2020, the WHO issued a warning of a potential decline in routine immunization rates owing to the COVID-19 pandemic.^5^ An estimated 23 million children did not receive basic vaccines. Likewise, in this study, compared with the mean total immunization visits during March–May 2019, a decline (−28.9%) was recorded in those during March 2020–May 2020 (lockdown period).

The decline in vaccination recorded in March 2020 was −32.1%, which was higher than that in April 2020 (−13.9%). This decline could be attributed to the effective implementation of the partial lockdown and social distancing strategies in the third week of March 2020 and the ease in regulations regarding lockdowns in mid-April, except for 2 residential areas mostly occupied by non-Kuwaiti nationals. The decline in vaccination visits and delivered doses was at its highest in May (−41.5%) owing to the surge in COVID-19 endemic cases and subsequent implementation of a complete lockdown, along with the closing of most ministries, authorities, and shops, except for those providing essential services, such as supermarkets and health facilities.

The results of this study were consistent with those of reports on substantial declines in scheduled immunizations across several countries in the early stages of the pandemic, including studies from the U.S. that reported an 18.5%–50% reduction from January 2020 to June 2020, compared with that in the pre‒COVID-19 period (2018–2019).^12^^,^^13^ In Spain, the reduction in the number of routine vaccinations administered to infants ranged from 8% to 20%.^14^ In Italy, early reports recorded that the number of vaccine doses administered in Rome was reduced by 16% over the first 10 weeks of lockdown.^15^ A large-scale immunization impact appraisal survey of 19 countries in Southeast Asian and Western Pacific regions conducted in June 2020 reported a disruption of vaccination coverage targets in most countries, except Australia and South Korea.^16^ In Karachi, Pakistan, the mean number of routine vaccination visits was reduced by 52.8% during the COVID-19 lockdown.^17^

Data from the Middle East are limited. However, the results of this study are consistent with reports from other countries in this region, which highlighted this drawback of immunization programs. In Riyadh, Saudi Arabia, the reduction in vaccination visits reached 72% in April 2020.^18^ In addition, delays in the delivery of recommended vaccines were reported in different parts of Saudi Arabia.^19^ A 40% decline in total childhood vaccine doses was recorded in Qatar in May 2020.^20^ In Egypt, 27.2% of children missed mandatory immunization owing to fear of COVID-19 infections.^21^ A 47% decline in public sector vaccine delivery was reported in Lebanon in March 2020 compared with the levels in 2019.^22^ Moreover, a survey of the vaccination status of pediatricians in Morocco revealed that most scheduled childhood vaccines were postponed during the initial pandemic stage.^23^

This reduction in immunization coverage raises concerns that a significant proportion of improvement in public health achieved by immunization will be lost. Vaccination prevents an estimated 2.7 million cases of measles, 2 million cases of neonatal tetanus, 1 million pertussis cases, 600,000 cases of poliomyelitis, and 300,000 diphtheria cases annually.^24^ A high rate of infant and child immunization is the major contributor to the eradication, elimination, or control of infectious diseases and their complications. This is achieved either by routine immunization or through supplementary immunization activities, providing direct protection and broader indirect protection through herd immunity.^25^

No distinguishable patterns in vaccination changes were identified in terms of age distribution. However, the highest decline was observed for vaccinations administered at age of 24 months (MMR2 and VV2) at −44.2%, followed by those administered at the age of 18 months (Penta4, PCV4, and OPV2) at −36.5% and at 1 year (MMR1 and VV1) at −28.8%. Likewise, in the U.S., the decline in measles vaccination (80%) was seemingly higher among children aged >24 months than among those aged ≤24 months (50%).^26^ This age-based difference in vaccination could be due to greater parental concern about the children during the first year of life than at later ages. During the early months of a child's life, parents tend to be more considerate and sensitive to their children's strengths and symptoms and alert regarding the need for prompt health management when necessary. It is possible that positive parenting behaviors such as preventing exposure to infection by increased compliance to vaccination decrease health risks in children, resulting in fewer illnesses and reducing the child's need for health care.^27^

The decline in vaccination in 2020 was higher among non-Kuwaiti children than among Kuwaiti children for all vaccine types, and the rebound increase later was higher among Kuwaiti children than among non-Kuwaiti children. This result can be attributed to the temporary departure of some non-Kuwaiti families to their native countries until the end of the pandemic and the easing of all restrictions, especially after the closure of schools and other educational institutions. Nevertheless, 2 areas largely inhabited by non-Kuwaiti residents were under complete lockdown and were secured by civil defense soldiers for >3 months. Although the health centers in these areas were open and operational for 24 hours daily, the local population believed that these centers were only open for emergencies. Previous literature indicates that ethnic or racial minorities are at a disproportionate risk for VPDs, and increasingly high levels of vaccination disparities exist among these groups.^28^ The gap in immunization coverage among various economic levels has been established earlier.^29^^,^^30^ This variation could be due to regional cultural differences affecting levels of compliance with health guidelines, level of concern regarding infection, differences in socioeconomic status,^17^ and existence of significant barriers while accessing healthcare services.^31^ In a study on MMR immunization in a primary care network in Ohio, children without insurance were found to be less likely to be immunized than children with private or governmental insurance.^32^

Disruptions in immunization services were probably due to interactions among multiple determinants. The COVID-19 pandemic, the resultant issuing of health warnings, and the implementation of strict preventive measures forged an atmosphere of fear among the public, leading to disruptions in all health services and consequently affecting vaccination coverage rates. Although health services were regularly offered regardless of the lockdowns, there was concern among the public about the lack of protection from the virus at healthcare facilities, along with a fear of contracting infections in waiting rooms and contact with infected persons.^14^^,^^15^^,^^33^ In addition, members of the public were unwilling to leave the security of their homes and experience transportation interruptions and restrictions on movement.^17^ Some individuals believed that healthcare providers were unavailable owing to limits on travel and transportation and redeployment to COVID-19 response recommendations and that there existed a lack of personal protective equipment owing to high demands from healthcare providers and the public.^8^^,^^17^ In addition, concerns regarding overburdening the overstretched health services played a role.^34^

Public health leaders have taken prompt action to bridge the gap in vaccine administration. Primary healthcare centers have implemented multiple risk alleviation measures, including the provision and use of personal protective equipment, implementing changes in preventive clinic workflow to minimize contact with positive cases, activation of preclinic telephone screening of patient calls to identify individuals with suspected COVID-19 infection before permitting entry to the clinic, enforced limitation on the number of people allowed in the preventive clinic waiting area, tracing of vaccine defaulters, and setting of new appointments for catch-up vaccination. In certain cases, mobile units were set up for active visits to households where children had missed their scheduled vaccinations. These efforts have helped to restore a certain measure of confidence in the safety of visiting healthcare centers for immunization. Consequently, vaccination rates began to increase in June 2020–August 2020.^35^

A +31.8% increase in the mean total visits during the reopening period (June–August 2020) compared with those during the lockdown period (March 2020–May 2020) compensated for the reduction in vaccinations during the lockdown. Similarly, data from Germany indicated that although immunization visits reduced in the early phase of the pandemic and lockdown and 20% of children and 40% of adults did not schedule catch-up appointments, most vaccinations were later completed.^36^ In addition, in the Netherlands, the number of infants receiving the first dose of the MMR vaccine was reduced by 6–14%. This decline was later addressed by a catch-up campaign.^37^ In the U.S., routine pediatric vaccinations were curtailed after a governmental emergency was asserted on March 13, 2020; however, the rate of vaccination for measles in infants aged <2 years has subsequently recovered since then.^26^

The immunization coverage for all antigens in Kuwait has been maintained at >98% for the last 2 decades. This rate was disrupted during the pandemic. Although the vaccination coverage increased in 2021, it did not attain the initial levels as in the corresponding period in 2019. Some vaccination rates remained lower than the prepandemic rates, as indicated by the annual data from the EPI department, showing a decline in immunization coverage for vaccines administered at the age of 6 months (Penta3, PCV3, and inactivated polio vaccine 3) and 2 years (VV2 and MMR2) (Appendix Table 2, available online). These results are similar to those reported by Langdon-Embry in New York, U.S.^38^ This may necessitate the implementation of further activities to promote the maintenance of scheduled vaccinations in these age groups, and catch-up vaccination may be needed to prevent VPD outbreaks.

### Limitations

We used aggregated data that were not differentiated in sociodemographic characteristics, hence associations between such variables and the outcome, which is missed vaccination visits, were difficult to determine. In addition, we could not explore the barriers affecting this disruption from the parent's perspective. Could it be that COVID-19 vaccine misinformation attitudes impacted vaccine acceptance and raised vaccine hesitancy in general, or could it be other factors that may have had an impact on promoting timely immunization uptake than the lockdowns and stay-at-home orders?

We did not include an analysis of vaccines given outside the age range for >24 months. Future studies need to monitor the increased number of immunization program defaulters by geography and should identify the possible residential districts and/or special groups that may need more intensive catch-up immunization campaigns to be carried out to prevent VPDs outbreaks.

We compared the disruption caused by the pandemic on the number of vaccination visits with pre‒COVID-19 vaccination visits but could not report the vaccination coverage rate changes during these periods.

## CONCLUSIONS

This study document a large negative impact of the COVID-19 pandemic on childhood vaccinations, with unprecedented declines in vaccination visits and vaccination coverage rates in Kuwait. Recoveries of vaccination visits were recorded in subsequent months, but they did not reach baseline levels in 2019. Therefore, the COVID-19 pandemic had negative impacts beyond direct viral infection, including the jeopardization of EPI services. Prolonged lockdown and isolation unfortunately led to a reluctance to commit to the immunization schedule. These findings may help guide public health policymakers to understand the impacts of the COVID-19 pandemic on preventive services and suggest the need to implement strategies to monitor the use of essential immunization services during any pandemic in the future.

## ETHICAL CONSIDERATION

This study was conducted in accordance with the declaration of Helsinki. The Ethics Committee for Medical Research at the Ministry of Health, State of Kuwait research provided approval for the study (identification number MOH/1822/2021) on October 5, 2021. The confidentiality of participants was secured by deidentifying all data included in the analysis. Data were kept in an encrypted file and saved on a computer, which was accessible to the principal investigator only.

## Appendix Supplementary materials

Image, application 1

Image, application 2
