# Supplementary material for: Impact of COVID-19 Pandemic on Routine Immunization in State of Kuwait: Short-Term Disruption With Rebound in Vaccination Utilization
Source: AJPM Focus. 2022 Sep 12;1(2):100031. doi: 10.1016/j.focus.2022.100031 (PMC9464581; doi:10.1016/j.focus.2022.100031)
Supplement: Supplementary file 1 [file mmc1.docx]

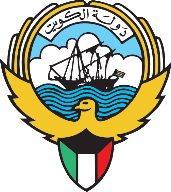


Ministry of Health – State of Kuwait

Kuwait Childhood Immunization Schedule 2019

| Age of Vaccination | Vaccine Type | Route of  Administration |
| --- | --- | --- |
| Pregnant mother | - 2 doses of Tetanus Toxoid at 5^Th^ & 7^th^ Month of 1^st^ Pregnancy. | - I.M |
| Within 24 hrs of birth | - 1^st^ Dose of Hepatitis B (HBV) | - I.M |
| End of 2^nd^ month | - 1^st^ Dose of IPV - 1^st^ Dose of DPT/HBV/Hib* - 1^st^ Dose of Pneumococcal (Ped) - 1^st^ Dose of Rota | - I.M - I.M - I.M - Oral |
| End of 3^rd^ month | - - BCG vaccine | - I.D. |
| End of 4^th^ month | - 2^nd^ Dose of IPV - 2^nd^ Dose of DPT/HBV/Hib* - 2^nd^ Dose of Pneumococcal (Ped) - 2^nd^ Dose of Rota | - I.M - I.M - I.M - Oral |
| End of 6^th^ month | - 3^rd^ Dose of IPV - 3^rd^ Dose of DPT/HBV/Hib* - 3^rd^ Dose of Pneumococcal (Ped) - 3^rd^ Dose Rota ** | - I.M - I.M - I.M - Oral |
| End of 12 months | - 1^st^ Dose of OPV - 1^st^ Dose MMR Vaccine - 1^st^ Dose Varicella Vaccine - Conjugate Meningitis | - Oral - S.C. - S.C. - I.M. |
| At 18^th^ month | - 2^nd^ Dose of OPV - Booster Dose of DPT/HBV/Hib* - Booster Dose Pneumococcal | - Oral - I.M - I.M |
| End of 2 years | - 2^nd^ Dose MMR Vaccine - 2^nd^ Dose Varicella Vaccine | - S.C. - S.C. |
| End of 3.5 years | - Booster Dose of OPV - Booster Dose of DPT | - Oral - I.M |
| 4-6 years (Preschool) | Before school admission every child should Checked or Immunization status for the appropriate vaccines as mentioned before | |
| 10-12 years  (at School) | - Booster Dose of MMR (for females only) - Booster Dose of Tetanus/Diphtheria. | - S.C - I.M |
| 16-18 years  (at School) | - Booster Dose of Tetanus/Diphtheria | - I.M |

*** Diphtheria Tetanus Pertussis Toxoid, Hepatitis B Vaccine, Haemophilus influenza b Vaccine.**

**** a third dose is needed when RV5 is used.**
