# Supplementary material for: Impact of COVID-19 Pandemic on Routine Immunization in State of Kuwait: Short-Term Disruption With Rebound in Vaccination Utilization
Source: AJPM Focus. 2022 Sep 12;1(2):100031. doi: 10.1016/j.focus.2022.100031 (PMC9464581; doi:10.1016/j.focus.2022.100031)
Supplement: Supplementary file 2 [file mmc2.docx]

| ***Appendix Table 2. Vaccination Coverage State of Kuwait from 2000 - 2021*** | | | | | | | | | | | | | | | | | | | | | | |
| --- | --- | --- | --- | --- | --- | --- | --- | --- | --- | --- | --- | --- | --- | --- | --- | --- | --- | --- | --- | --- | --- | --- |
| **Vaccine** | **DTP/DTP-HIB / DTP-HIP-HEPATITIS 3** | **PNEUMOCOCCAL 3** | **OPV3** | **HEPATITIS 3** | **MMR1** | **MMR2** | **MENINGOCOCCAL Polysaccharide** | **MENINGOCOCCAL Conjugated** | **BCG** | **IPV1** | **IPV2** | **IPV3** | **OPV1** | **OPV2** | **OPV3** | **VV1** | **VV2** | **Rota1** | **Rota2** | **Rota3** | **PCV4** | **Penta 4** |
| **AGE** | **6 months** | **6 months** | **6 months** | **6 months** | **12 months** | **24months** | **24 months** | **12 months** | **newborn** | **2 months** | **4 months** | **6 months** | **12 months** | **18 months** | **3.6 Y** | **12 months** | **24months** | **2 months** | **4 months** | **6 months** | **18 months** | **18 months** |
| **2000** | **97.7** |  | **98.43** | **96.8** | **96.83** |  | **94** |  |  |  |  |  |  |  |  |  |  |  |  |  |  |  |
| **2001** | **97.6** |  | **99** | **97.6** | **98.5** |  | **99** |  |  |  |  |  |  |  |  |  |  |  |  |  |  |  |
| **2002** | **99** |  | **99** | **99** | **97.3** |  | **99** |  |  |  |  |  |  |  |  |  |  |  |  |  |  |  |
| **2003** | **99** |  | **99** | **99** | **97.4** |  | **99** |  |  |  |  |  |  |  |  |  |  |  |  |  |  |  |
| **2004** | **97.6** |  | **97.6** | **97.8** | **97.3** |  | **99** |  |  |  |  |  |  |  |  |  |  |  |  |  |  |  |
| **2005** | **99** |  | **99** | **99** | **99** |  | **99** |  |  |  |  |  |  |  |  |  |  |  |  |  |  |  |
| **2006** | **99** |  | **99** | **99** | **99** |  | **99** |  | **84** |  |  |  |  |  |  |  |  |  |  |  |  |  |
| **2007** | **99** | **88.5** | **99** | **99** | **99** |  | **99** |  | **94** |  |  |  |  |  |  |  |  |  |  |  | **88.5** | **99** |
| **2008** | **99** | **99** | **99** | **99** | **99** | **86.2** | **99** |  | **99** |  |  |  |  |  |  |  |  |  |  |  | **99** | **99** |
| **2009** | **99** | **99** | **99** | **99** | **99** | **93.5** | **99** |  | **99** |  |  |  |  |  |  |  |  |  |  |  | **99** | **99** |
| **2010** | **98** | **98** | **98** | **99** | **99** | **99** | **99** |  | **99** |  |  |  |  |  |  |  |  |  |  |  | **98** | **99** |
| **2011** | **99.2** | **99.7** | **99.1** | **99.2** | **99** | **99** | **99** |  | **99** |  |  |  |  |  |  |  |  |  |  |  | **99.7** | **99.2** |
| **2012** | **99** | **99** | **99** | **99** | **99** | **99** | **99** |  | **98** |  |  |  |  |  |  |  |  |  |  |  | **99** | **99** |
| **2013** | **94.8** | **93.7** | **94.8** | **95.97** | **93.89** | **86.4** | **86.9** |  | **99.1** |  |  |  |  |  |  |  |  |  |  |  | **93.7** | **95.97** |
| **2014** | **97** | **98** | **98** | **97** | **91** | **93** | **91.2** |  | **98** |  |  |  |  |  |  |  |  |  |  |  | **98** | **97** |
| **2015** | **99** | **99** | **99** | **99** | **93.2** | **95.5** | **95.4** |  | **99** |  |  |  |  |  |  |  |  |  |  |  | **99** | **99** |
| **2016** | **99** | **99** | **99** | **99.7** | **99** | **98.7** | **98.5** |  | **95.6** |  |  |  |  |  |  |  |  |  |  |  | **99** | **89** |
| **2017** | **99** | **99** | **99** | **99** | **99** | **99** |  | **99** | **99** |  |  |  |  |  |  |  |  |  |  |  | **99** | **95** |
| **2018** | **94** | **93** | **99** | **94** | **99** | **97** |  | **99** | **98** |  |  |  |  |  |  |  |  | **94** | **71** | **60** | **93** | **99** |
| **2019** | **99** | **99** |  | **99** | **99** | **99** |  | **99** | **99** | **99*** | **61*** | **62*** | **91** | **92** | **89** | **99** | **99** | **97** | **91** | **80** | **99** | **99** |
| **2020** | **95** | **95** |  | **95** | **99** | **86** |  | **98** | **99** | **99** | **96** | **96** | **99** | **99** | **90** | **99** | **87** | **96** | **92** | **80** | **99** | **99** |
| **2021** | **94** | **94** |  | **94** | **94** | **84** |  | **99** | **99** | **99** | **96** | **94** | **85** | **88** | **87** | **93** | **84** | **97** | **89** | **75** | **96** | **96** |
| MMR2 at 4-6 years with school health department was: 2005 = 77% , 2006 = 95.5% , 2007= 90.4% | | | | | | | | | | | | | | | | | | | | | | |
| IPV introduced April 2019, More than Half of cohort vaccinate with IPV, The remaining continued on OPV so total vaccination coverage with either OPV or IPV at 6 month of age is 99% | | | | | | | | | | | | | | | | | | | | | | |
